# Efficient Computation of Free Energy Surfaces of Diels–Alder Reactions in Explicit Solvent at Ab Initio QM/MM Level

**DOI:** 10.3390/molecules23102487

**Published:** 2018-09-28

**Authors:** Pengfei Li, Fengjiao Liu, Xiangyu Jia, Yihan Shao, Wenxin Hu, Jun Zheng, Ye Mei

**Affiliations:** 1 State Key Laboratory of Precision Spectroscopy, School of Physics and Materials Science, East China Normal University, Shanghai 200062, China; lipengfei_mail@126.com (P.L.); fjliu208@gmail.com (F.L.); xj6@nyu.edu (X.J.); 2 Department of Chemistry and Biochemistry, University of Oklahoma, Norman, OK 73019, USA; yihan.shao@ou.edu; 3 The Computer Center, School of Computer Science and Software Engineering, East China Normal University, Shanghai 200062, China; jzheng@cc.ecnu.edu.cn; 4 NYU-ECNU Center for Computational Chemistry at NYU Shanghai, Shanghai 200062, China

**Keywords:** Diels–Alder reaction, free energy surface, ab initio, reference-potential method, umbrella sampling

## Abstract

For Diels–Alder (DA) reactions in solution, an accurate and converged free energy (FE) surface at ab initio (*ai*) quantum mechanical/molecular mechanical (QM/MM) level is imperative for the understanding of reaction mechanism. However, this computation is still far too expensive. In a previous work, we proposed a new method termed MBAR+wTP, with which the computation of the *ai* FE profile can be accelerated by several orders of magnitude via a three-step procedure: (I) an umbrella sampling (US) using a semi-empirical (SE) QM/MM Hamiltonian is performed; (II) the FE profile is generated using the Multistate Bennett Acceptance Ratio (MBAR) analysis; and (III) a weighted Thermodynamic Perturbation (wTP) from the SE Hamiltonian to the *ai* Hamiltonian is performed to obtain the ai QM/MM FE profile using weight factors from the MBAR analysis. In this work, this method is extended to the calculations of two-dimensional FE surfaces of two Diels–Alder reactions of cyclopentadiene with either acrylonitrile or 1-4-naphthoquinone at *ai* QM/MM level. The accurate activation free energies at the *ai* QM/MM level, which are much closer to the experimental measurements than those calculated by other methods, indicate that this MBAR+wTP method can be applied in the studies of complex reactions in condensed phase with much-enhanced efficiency.

## 1. Introduction

The Diels–Alder (DA) reactions are one category of organic chemical reactions (specifically, a [4+2] cycloaddition) between a conjugated diene and a dienophile, which involve a dual carbon–carbon bond-forming process. Among them, the DA reactions of cyclopentadiene (CP) with acrylonitrile (ACR) or 1-4-naphthoquinone (NAP) have attracted much attention from experimental [1,2,3] and computational scientists [4,5,6,7,8]. It has been observed that the reaction rate is very sensitive to the solvent [2,3,5,6,7,8]. Therefore, the solvation effect should be explicitly considered in order to unveil the reaction mechanism, in which the polarization effect and the reorganization of the solvent molecules may play a critical role.

Hybrid quantum mechanical/molecular mechanical (QM/MM) method, which was proposed by Warshel and Levitt in 1976 [9], is now a mature method that can be used to study chemical reactions taking place in condensed phase such as aqueous solution or enzymatic environment [10,11,12,13]. In this approach, only reactive region is treated quantum mechanically, and the remaining part is described by molecular mechanical force field. However, when the reaction barrier is much larger than $k_{B}T$, which is pervasive for reactions under mild condition, a direct ab initio (*ai*) QM/MM simulation is still notoriously time consuming, if feasible at all. Despite the continuous development in computer technology and enhanced sampling methods, investigation of reaction mechanism in solution or enzymes using direct *ai* QM/MM simulations is still a daunting task.

To reduce the computational expense, Jorgenson et al. proposed a new approach for the calculations of free energy changes in chemical reactions in solution by combining gas phase QM calculations with free energy simulations (QM-FE method) [14,15,16]. Kollman et al. extended this method to the studies of enzymatic reactions [17]. However, in the QM-FE approach, the QM Hamiltonian and the MM Hamiltonian are separated, which is not a rigorous QM/MM approach. In other words, the impact of the solvent or the enzyme environment on the electronic structure of the QM region is not considered. However, solvent or enzyme environment often has a remarkable impact on the reaction process. Zhang and Yang [18] were motivated by the QM-FE approach and developed a more practical method, which combines *ai* QM/MM calculations with free energy perturbation (FEP) [19,20]. Later, Thiel et al. named this method “QM/MM-FEP” [21]. In this method, an efficient iterative optimization procedure was developed to determine the optimized structures and the minimum energy paths for a large-sized system on an *ai* QM/MM potential energy surface. However, this still requires arduous computations.

To further reduce the computational expense, Jorgensen et al. used semi-empirical methods such as AM1 and PDDG/PM3 in the free energy calculations of these DA reactions [6,7,8], followed by some high-level correction to the stationary points in the reaction path including the reactant, the product and the transition state (TS) [7]. They found that the solvent-sensitivity originated from a significant nonhydrophobic component stemming from enhanced polarization of the transition state, which leads to strengthened hydrogen bonds [6,7,22,23]. They also found that the DA reaction between CP and ACR was an asynchronous and concerted process [4], while that between CP and NAP has a synchronous and concerted feature [6,7]. This observation was evidenced in a two-dimensional free energy landscape for these DA reactions, and a one-dimensional reaction coordinate for these reactions would lead to potential artifacts and uncertainty in the locations of transition states, which in the end leads to an ambiguous reaction mechanism [6,7]. Although semi-empirical (SE) QM/MM calculations have been widely used [12,24,25,26,27,28,29], unfortunately, these semi-empirical methods may lead to large errors in the results due to the approximations adopted. Thus, high-level quantum mechanical methods at ab initio levels are necessary for a reliable depiction of electron redistribution during the reaction, which can be critical to the energetics such as activation and reaction free energies.

Based on the idea of probability-reweighting, Gao developed a method termed the dual-Hamiltonian method, also known as the reference-potential method, and applied it in a study of hydration free energy [30,31]. Utilizing an empirical valence bond (EVB) method [32,33] as the reference-potential, Warshel et al. developed a dual-Hamiltonian approach for calculating the free energy (FE) profiles of chemical or enzymatic reactions, from which an *ai* FE profile can be obtained with much lower computational expense than a direct approach [26,34,35,36]. Rod and Ryde calculated the activation free energy of a methyl transfer reaction in enzyme using the dual-Hamiltonian approach where the free energy was found to be overestimated [37]. Recently, Jia et al. used the dual-Hamiltonian approach to calculate the solvation free energies of the molecules in the SAMPL4 competition by an alchemical decoupling method, which yielded the globally minimal variance for the QM/MM free energies [38]. Liu et al. used this dual-Hamiltonian approach to calculate protein–ligand binding affinity at an *ai* QM/MM level [39].

In our previous work [40], in the spirit of reference-potential method, a new method termed MBAR+wTP was proposed to obtain the *ai* QM/MM FE profiles with much less computational expense. In this method, a weighted thermodynamic perturbation (wTP) [19] correction is applied to the semi-empirical profile, which is generated by the Multistate Bennett Acceptance Ratio (MBAR) [41,42] analysis of the trajectories from umbrella sampling (US) [43]. The raw *ai* QM/MM FE profile was then smoothed via Gaussian process regression [44]. This MBAR+wTP method had been validated by calculating the FE profiles of one quasi-chemical reaction and three chemical reactions in aqueous solution. The results showed that even if the SE FE profiles deviated from the *ai* ones by several kcal/mol in terms of activation free energy and reaction free energy, after the SE-to-*ai* correction the FE profiles agree much better with the direct QM/MM simulated ones with errors below 1 kcal/mol.

In this work, we applied the MBAR+wTP method to calculate the FE surfaces of two Diels–Alder reactions of cyclopentadiene with either acrylonitrile or 1-4-naphthoquinone as mentioned above and investigated the applicability of this method to the study of reactions with two-dimensional (2D) reaction coordinates (RC).

## 2. Methods

### 2.1. Multistate Bennett Acceptance Ratio and Weighted Thermodynamic Perturbation (MBAR+wTP) Method

In our previous work [40], all the reactions studied were characterized by a one-dimensional generalized reaction coordinate. It is well-known that the reference-potential method suffers from numerical difficulty when the reference Hamiltonian and the target Hamiltonian have no significant overlap in phase space. With the increasing complexity of the molecule, this difficulty becomes more and more severe, and the applicability of this method is challenged. This work aims to investigate the applicability of this method to the calculations of the *ai* FE surfaces of two Diels–Alder reactions with two-dimensional reaction coordinates.

The two-dimensional reaction coordinate is denoted by $\eta \equiv (\eta_{1}\left(x\right),\eta_{2}\left(x\right))$, where $\eta_{1}\left(x\right)$ and $\eta_{2}\left(x\right)$ are functions of the collective atomic coordinates x. In two-dimensional US simulations [43], two harmonic restraining potentials $W_{i}^{1}\left(\eta_{1}\right)=\frac{1}{2}k_{1,i}{\left(\eta_{1}-\eta_{1,i}\right)}^{2}$ and $W_{i}^{2}\left(\eta_{2}\right)=\frac{1}{2}k_{2,i}{\left(\eta_{2}-\eta_{2,i}\right)}^{2}$ are added to the original potential energy surface $U_{0}\left(x\right)$ of the system, where $\eta_{1,i}$ and $\eta_{2,i}$ are the target values of RC, and $k_{1,i}$ and $k_{2,i}$ are the strengths of the restraints on the first and second dimensions, respectively, in the *i*th biased window. A set of two-dimensional biased window simulations indexed by *i* (i=1,2,⋯,S) are carried out with the potential energy surfaces $U_{i}^{\left(b\right)}\left(x\right)=U_{0}\left(x\right)+W_{i}\left(\eta \right)$, where $W_{i}\left(\eta \right)=W_{i}^{1}\left(\eta_{1}\right)+W_{i}^{2}\left(\eta_{2}\right)$ is the restraint potential. The trajectories are then post-processed using the MBAR method to obtain the unbiased thermodynamic properties on the original potential energy surface $U_{0}\left(x\right)$. The nonlinear equations are solved using the pyMBAR program.

The ensemble average of any physical operator $\hat{O}$ under Hamiltonian H can always be computed via

(1) $${\langle \hat{O}\rangle }_{H}=\sum_{l}\omega_{H}^{l}O_{l},$$

where $O_{l}$ is the value of the operator $\hat{O}$ for configuration *l*, $\omega_{H}^{l}$ is the *normalized* weight of this configuration under Hamiltonian H, and the summation is over all the configurations. Strictly speaking, we should use thermodynamic state instead of Hamiltonian. The ensemble average is also a function of other macroscopic thermodynamic parameters such as temperature. In this work, the only difference between two thermodynamic states is the Hamiltonian, while the temperature is kept the same. Therefore, we use Hamiltonians to differentiate the thermodynamic states. In the reference-potential method, simulations are carried out under an inexpensive Hamiltonian L, sometimes with biasing potential *W* as, for instance, in umbrella sampling. With the samples harvested in the simulations, ensemble averages under an expensive Hamiltonian H can be calculated via energy reweighting, which will be explained in the following. In this way, the expensive sampling under Hamiltonian H is avoided.

Suppose we have carried out an umbrella sampling simulation with *S* windows in total. For the *i*th window, the potential is $U_{i}^{\left(b\right)}\left(x\right)=U_{L}\left(x\right)+W_{i}\left(\eta \right)$, with $U_{L}\left(x\right)$ being the unbiased potential and $W_{i}\left(\eta \right)$ the biasing potential. For the *l*th configuration from the *i*th window $x_{i,l}$, the weight under the unbiased Hamiltonian L can be written as [40,42]

(2) $$\begin{array}{cc} w_{L}\left(x_{i,l}\right)= & \frac{e^{-\beta \left[U_{L}\left(x_{i,l}\right)-f_{L}\right]}}{\sum_{k=1}^{S}N_{k}e^{-\beta \left[U_{k}^{\left(b\right)}\left(x_{i,l}\right)-f_{k}^{\left(b\right)}\right]}}, \end{array}$$

where $f_{k}^{\left(b\right)}$ is the free energy of the biased window *k* and β is the reciprocal of the thermodynamic temperature. $f_{k}^{\left(b\right)}$ can be obtained by iteratively solving the core equations in the MBAR method [40,41,42].

(3) $$\begin{array}{cc} e^{-\beta f_{k}^{\left(b\right)}}= & \sum_{i=1}^{S}\sum_{l=1}^{N_{i}}\frac{e^{-\beta \left[W_{k}\left(x_{i,l}\right)\right]}}{\sum_{t=1}^{S}N_{t}e^{-\beta \left[W_{t}\left(x_{i,l}\right)-f_{t}^{\left(b\right)}\right]}}. \end{array}$$

The numerator $e^{\beta f_{L}}$ in Equation (2) is constant for a given Hamiltonian L, which can be canceled during the normalization. By eliminating $U_{L}\left(x\right)$ from both the numerator and denominator in Equation (2), the *unnormalized* weight of sample $x_{i,l}$ under the unbiased Hamiltonian L is

(4) $$\begin{array}{cc} w_{L}^{0}\left(x_{i,l}\right)= & \frac{1}{\sum_{k=1}^{S}N_{k}e^{-\beta \left[W_{k}\left(x_{i,l}\right)-f_{k}^{\left(b\right)}\right]}}, \end{array}$$

in which the superscript 0 is added to emphasize that this is the unbiased weight. The unbiased two-dimensional FE surface, with the reaction coordinate η represented as a vector, can thus be expressed as

(5) $$\begin{array}{cc} F_{L}\left(\eta \right)= & -\beta^{-1}\ln\sum_{i=1}^{S}\sum_{l=1}^{N_{i}}w_{L}^{0}\left(x_{i,l}\right)\delta \left(\eta (x_{i,l})-\eta \right), \end{array}$$

in which the delta function picks out the samples falling into bin η.

The variance of the estimated free energy difference between bin $\eta_{i}$ and bin $\eta_{j}$ can be obtained by

(6) $$\begin{array}{c} \delta^{2}\Delta F_{L}\left(\eta_{ij}\right)=\Theta_{ii}-2\Theta_{ij}+\Theta_{jj}, \end{array}$$

where the covariance matrix is obtained from Equation D8 in Ref. [41] by

(7) $$\begin{array}{cc} \Theta = & {\left[{\left(W^{T}W\right)}^{-1}-N+1_{S+M}1_{S+M}^{T}/N\right]}^{-1}, \end{array}$$

where $N=diag(N_{1},N_{2},\cdots ,N_{S},0_{1},0_{2},\cdots ,0_{M})$; *M* is the number of bins; $N_{1},N_{2},\cdots ,N_{S}$ are, respectively, the number of samples in the 1st, 2nd, ..., *S*th simulation window; *N* is the total number of samples collected from all the simulation windows; and W is a matrix with a dimension of N×(S+M), whose elements are depicted in detail in Ref. [40].

By combining the binned configurations from the two-dimensional US simulations with $U_{i}^{\left(b\right)}\left(\eta \right)=U_{L}\left(x\right)+W_{i}\left(\eta \right)$ and their weights from the MBAR analysis, the FE surface at an arbitrary high level Hamiltonian H can be calculated by wTP. For a certain two-dimensional histogram bin, the free energy difference between the low-level and the high-level Hamiltonians can be obtained via weighted TP as

(8) $$\begin{array}{cc} \Delta F(\eta )= & -\frac{1}{\beta }\ln\frac{\sum_{i=1}^{S}\sum_{l=1}^{N_{i}}w_{L}^{0}\left(x_{i,l}\right)\delta \left(\eta (x_{i,l})-\eta \right)e^{-\beta \left[U_{H}\left(x_{i,l}\right)-U_{L}\left(x_{i,l}\right)\right]}}{\sum_{i=1}^{S}\sum_{l=1}^{N_{i}}w_{L}^{0}\left(x_{i,l}\right)\delta \left(\eta (x_{i,l})-\eta \right)}, \end{array}$$

where the subscripts H and L denote the high-level and the low-level Hamiltonians, respectively. Again, the delta function picks out the samples falling into bin η. Because the two-dimensional US samplings were performed at the low-level Hamiltonian, the FE surface $F_{L}\left(\eta \right)$ corresponding to this low-level Hamiltonian L can be obtained via Equation (5). Then, the FE surface of the high-level Hamiltonian H can be calculated by

(9) $$F_{H}\left(\eta \right)=F_{L}\left(\eta \right)+\Delta F\left(\eta \right).$$

It is noted that any physical quantity ${\langle O\left(\eta \right)\rangle }_{H}$ at the high-level Hamiltonian H can be obtained via the reweighting method

(10) $$\begin{array}{cc} {\langle O\left(\eta \right)\rangle }_{H}= & \frac{\sum_{i=1}^{S}\sum_{l=1}^{N_{i}}O\left(x_{i,l}\right)w_{L}^{0}\left(x_{i,l}\right)\delta \left(\eta (x_{i,l})-\eta \right)e^{-\beta \left[U_{H}\left(x_{i,l}\right)-U_{L}\left(x_{i,l}\right)\right]}}{\sum_{i=1}^{S}\sum_{l=1}^{N_{i}}w_{L}^{0}\left(x_{i,l}\right)\delta \left(\eta (x_{i,l})-\eta \right)e^{-\beta \left[U_{H}\left(x_{i,l}\right)-U_{L}\left(x_{i,l}\right)\right]}}, \end{array}$$

and its variance is computed via

(11) $$\begin{array}{cc} \delta^{2}{\langle O\left(\eta \right)\rangle }_{H}= & \frac{\sum_{i=1}^{S}\sum_{l=1}^{N_{i}}O^{2}\left(x_{i,l}\right)w_{L}^{0}\left(x_{i,l}\right)\delta \left(\eta (x_{i,l})-\eta \right)e^{-\beta \left[U_{H}\left(x_{i,l}\right)-U_{L}\left(x_{i,l}\right)\right]}}{\sum_{i=1}^{S}\sum_{l=1}^{N_{i}}w_{L}^{0}\left(x_{i,l}\right)\delta \left(\eta (x_{i,l})-\eta \right)e^{-\beta \left[U_{H}\left(x_{i,l}\right)-U_{L}\left(x_{i,l}\right)\right]}}\\ & -{\left(\frac{\sum_{i=1}^{S}\sum_{l=1}^{N_{i}}O\left(x_{i,l}\right)w_{L}^{0}\left(x_{i,l}\right)\delta \left(\eta (x_{i,l})-\eta \right)e^{-\beta \left[U_{H}\left(x_{i,l}\right)-U_{L}\left(x_{i,l}\right)\right]}}{\sum_{i=1}^{S}\sum_{l=1}^{N_{i}}w_{L}^{0}\left(x_{i,l}\right)\delta \left(\eta (x_{i,l})-\eta \right)e^{-\beta \left[U_{H}\left(x_{i,l}\right)-U_{L}\left(x_{i,l}\right)\right]}}\right)}^{2}. \end{array}$$

### 2.2. Gaussian Process Regression for FE Surfaces Smoothing

Following the same way as in our previous work [40], a nearly model-free method called Gaussian processes regression (GPR) [44] was utilized to smooth the FE surface after the wTP correction, removing the statistical noise in the wTP correction process as shown in Figure S3. Given a set of observations $\left\{F_{1},F_{2},\ldots ,F_{n}\right\}$, it can be viewed as a single sample from a Gaussian distribution with *n* variates. Here, due to the two-dimensional reaction coordinates of the reactions studied in this work, each variate has two features which can be labeled by vector η. Since the observations are noisy, each observation *F* is related to an underlying function f(η) through a Gaussian noise model

(12) $$\begin{array}{c} F=f\left(\eta \right)+N\left(0,\sigma_{n}^{2}\right). \end{array}$$

Then, the covariance function kernel *k* was defined using the squared exponential as

(13) $$\begin{array}{c} k\left(\eta ,\eta^{{}^{\prime }}\right)=\sigma_{f}^{2}\exp\left[\frac{-{\left(\eta -\eta^{{}^{\prime }}\right)}^{2}}{2l^{2}}\right]+\alpha \sigma_{n}^{2}\delta \left(\eta ,\eta^{{}^{\prime }}\right), \end{array}$$

where *l* is the length-scale and $\sigma_{f}^{2}$ is the signal variance, $\delta (\eta ,\eta^{{}^{\prime }})$ is the Kronecker delta function and $\sigma_{n}^{2}$ is the noise variance, which was set to the reciprocal of the exponential of the reweighting entropy [45] value ($e^{-S}$) corresponding to each observation in this work as done in our previous work [40]. The “hyperparameters” $\left\{l,\sigma_{f},\alpha \right\}$ are optimized to maximize the likelihood of the observations. For any point $\eta_{*}$ along the reaction coordinate, the free energy can be calculated, with the existence of *n* training data $\{\eta_{i},f\left(\eta_{i}\right)\},\;i=1,2,\cdots ,n$, via

(14) $$F\left(\eta_{*}\right)=k_{*}^{T}K^{-1}\left(\eta ,\eta \right)f\left(\eta \right),$$

and its variance via

(15) $$\delta^{2}F\left(\eta_{*}\right)=k\left(\eta_{*},\eta_{*}\right)-k_{*}^{T}K^{-1}\left(\eta ,\eta \right)k_{*},$$

where

(16) $$K=\left[\begin{array}{cccc} k(\eta_{1},\eta_{1}) & k(\eta_{1},\eta_{2}) & \cdots & k(\eta_{1},\eta_{n})\\ k(\eta_{2},\eta_{1}) & k(\eta_{2},\eta_{2}) & \cdots & k(\eta_{2},\eta_{n})\\ \vdots & \vdots & \ddots & \vdots \\ k(\eta_{n},\eta_{1}) & k(\eta_{n},\eta_{2}) & \cdots & k(\eta_{n},\eta_{n}) \end{array}\right],$$

(17) $$k_{*}={\left[\begin{array}{cccc} k(\eta_{*},\eta_{1}) & k(\eta_{*},\eta_{2}) & \cdots & k(\eta_{*},\eta_{n}) \end{array}\right]}^{T}.$$

and

(18) $$f\left(\eta \right)={\left[\begin{array}{cccc} f(\eta_{1}) & f(\eta_{2}) & \cdots & f(\eta_{n}) \end{array}\right]}^{T}.$$

Gaussian process regression was performed by using the scikit-learn package [46].

### 2.3. Locating the Transition State on the Free Energy Surface

The transition state is located on the smoothed free energy surface by satisfying two conditions

(19) $$\frac{\partial F}{\partial \eta_{i}}=0$$

and the Hessian $\partial^{2}F/\partial \eta_{i}\partial \eta_{j},\;i,j\in \left\{1,2\right\}$ has one positive and one negative eigenvalues, where F and $\eta_{i}$ are the free energy and the reaction coordinate, respectively. The gradient and the Hessian were computed using the finite central-difference method.

### 2.4. Solvent-Assisted Charge Transfer on the Transition State

**Figure 1:**
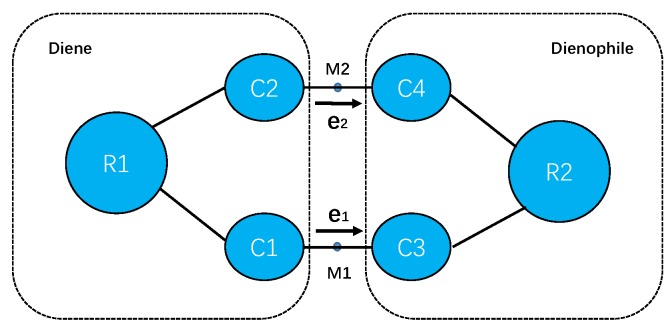
The diagram representation of active sites for Diels–Alder reactions in this work, where C1, C2, C3 and C4 are the four atoms shown in Figure 2, and the remaining atoms in diene are grouped together as R1, and those in dienophile as R2. The unit vector ${\vec{e}}_{1}$ points from atom C1 to atom C3 and the unit vector ${\vec{e}}_{2}$ points from atom C2 to atom C4. M1 and M2 are the two middle points of C1–C3 and C2–C4 bonds, on which the solvent electric fields are computed.

The solvent-assisted charge transfer on the transition state can be approximately delineated as interactions between the transferred charges within the newly formed C–C bonds generating a local dipole and the electric field on the middle point of the bonds (M1 or M2) contributed from all the solvent molecules as depicted in Figure 1. The electric field $\vec{E}$ on point *j* is a sum over the contributions from all the solvent molecules with atomic partial charge $q_{i}$ by

(20) $$\begin{array}{c} {\vec{E}}_{j}=\sum_{i}\frac{q_{i}\left({\vec{r}}_{j}-{\vec{r}}_{i}\right)}{{\left|{\vec{r}}_{j}-{\vec{r}}_{i}\right|}^{3}}, \end{array}$$

where ${\vec{r}}_{i}$ is the coordinate of atom *i* from solvent molecules, ${\vec{r}}_{j}$ is the coordinates of the middle points of newly formed C-C bonds (M1 or M2) as shown in Figure 1. The projections of the electric fields along the unit vectors ${\vec{e}}_{1}$ and ${\vec{e}}_{2}$, respectively, may facilitate or impede the charge transfer between the reactants. The ensemble averages of these physical quantities are computed via Equation (10) by substituting $\hat{O}$ with the corresponding operators. The interaction between the local dipole moment and the solvent-generated electric field can be computed via

(21) $$\Delta E=-0.5\left(\delta q_{2}-\delta q_{1}\right)\times l_{C-C}\times \hat{E},$$

where $\delta q_{1}$ and $\delta q_{2}$ are the changes of the atomic Mulliken charge from the reactant to the transition state for $C_{1}$(or $C_{2}$) and $C_{3}$ (or $C_{4}$), $l_{C-C}$ is the bond length of $C_{1}-C_{3}$ (or $C_{2}-C_{4}$), and $\hat{E}$ is the projection of the solvent-generated electric field along the $C_{1}-C_{3}$ (or $C_{2}-C_{4}$) bond.

### 2.5. Gibbs Free Energies in Implicit Water Solvent

To save computational cost, implicit solvent model is frequently used during the calculations of stationary structures, activation free energy and reaction free energy. In this method, instead of an ensemble of structures, a unique structure is used to represent each of the stationary points (such as reactant, transition state, and product), thus avoiding expensive sampling in the phase space. Reaction free energy and activation free energy can be computed under the rigid-rotor/harmonic-oscillator (RRHO) approximation via frequency analyses for the reactant, transition state and product. In this work, the calculations were carried out at the same ab initio levels as in explicit solvent (B3LYP/6-31G(d) or B3LYP-D3/6-31G(d) level), and the integral equation formalism of the polarizable continuous solvent model (IEF-PCM) was adopted for the solvation effect. In addition, we also chose a higher level *ai* method (MP2/6-311 + g(2d,p)/IEF-PCM), which is similar to that used in Ref. [7] by Jorgensen et al. For the optimization of the transition structures, the option opt = QST3 implemented in Gaussian 16 [47] was used, which requires three molecular specifications corresponding to the reactant, the product, and an initial guess for the transition state structure. All the transition state structures are verified via intrinsic reaction coordinate (IRC) analysis, in which both the reactant and the product can be identified along the IRC path starting from transition state structure.

### 2.6. Molecular Dynamics Simulations

**Figure 2:**
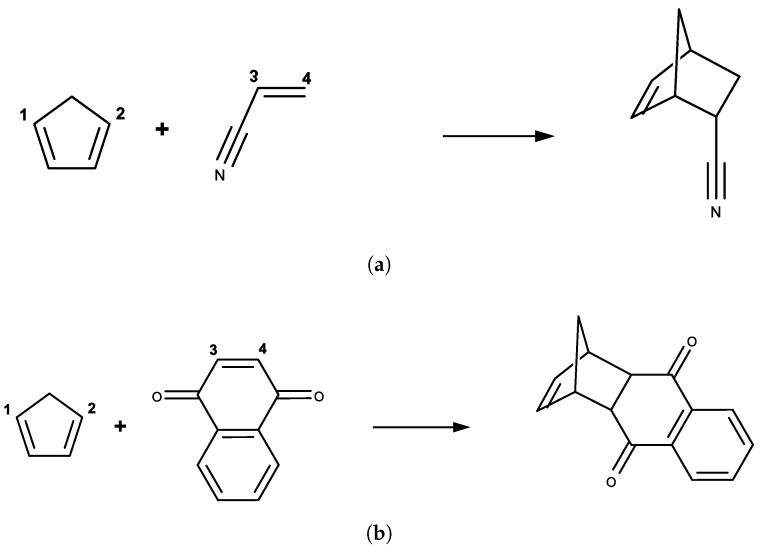
Diels–Alder reactions between: (**a**) cyclopentadiene and acrylonitrile; and (**b**) cyclopentadiene and 1-4-naphthoquinone, where $\eta_{1}=d_{C1C3}$ and $\eta_{2}=d_{C2C4}$ were chosen as the two-dimensional reaction coordinates.

Two Diels–Alder reactions of cyclopentadiene with either acrylonitrile or 1-4-naphthoquinone were studied in this work, which are shown in Figure 2. Only the reactant molecules including cyclopentadiene and either acrylonitrile or 1-4-naphthoquinone were defined as the semi-empirical QM (SE QM) or the QM region, and the remaining of the system (including only the water molecules) were defined as the MM region. A TIP3P water sphere with a radius of 25 Å was added to solvate the reactive molecules centering on the heavy atom closest to the center-of-mass of the QM regions and was restrained by a soft half-harmonic potential with a force constant of 10 kcal/mol/Å${}^{2}$ to prevent evaporation, as done by Thiel et al. [48]. There were 2007 water molecules for the ACR system and 1996 water molecules for the NAP system. The nonbonded interactions were fully considered without a truncation and the general AMBER force field [49] was assigned to the solute molecules. PM6 was used as the low-level Hamiltonian, and B3LYP(-D3)/6-31G(d) was chosen as the high-level QM Hamiltonian. Here, the electrostatic embedding scheme was used to explicitly take into account the polarization effect from the MM region on the QM region. The two-dimensional umbrella sampling simulations were performed at PM6/MM level. The indirect FE surfaces at B3LYP(-D3)/6-31G(d)/MM level was computed by reweighting from the PM6/MM level.

The endo addition mode for these reactions was chosen, because it corresponds to the preferred transition state from ab initio calculations [4,7] and experimental stereoselectivity preferences [1,5]. The $\eta_{1}=d_{C1C3}$ and $\eta_{2}=d_{C2C4}$ were chosen as two-dimensional reaction coordinates in both cases. Two-dimensional umbrella samplings were conducted centering on $\eta \equiv (\eta_{1},\eta_{2})$ ranged from 1.50 to 4.00 Å with increments of 0.05 Å for each dimension. The reactant state in both cases was defined as $\eta_{1}$ = 4.00 Å and $\eta_{2}$ = 4.00 Å, where the FE surfaces were rather flat in the vicinity. To reduce the computational cost, only the important region on the FE surface was sampled.

For each two-dimensional US window under the PM6/MM Hamiltonian, the system was energy-optimized for 500 steps using the steepest decent optimization method followed by 500 steps of the conjugate gradient method with the solute molecules restrained. Then, the same optimization procedure continued with the restraint removed. The system was heated up to 298.15 K in 50 ps and was equilibrated for 100 ps. A 1-ns production molecular dynamics (MD) simulation was performed for each window. The integration time step was set to 1 fs and the configurations were saved every 1 ps. The temperature was regulated at 298.15 K with the Andersen temperature coupling scheme [50]. Then, single point energies under the PM6/MM and B3LYP/6-31G(d)/MM Hamiltonians for the one thousand configurations saved in each US simulation were calculated and taken into the TP reweighting calculations. All the simulations were performed by the AmberTools 17 program package [51], and the QM/MM calculations were carried out by interfacing with Gaussian 16 package [47].

## 3. Results and Discussion

In the MBAR+wTP method, there are two key factors that are critical to the reliability of the results. The first one is the similarity between the Hamiltonians for neighboring windows in the two-dimensional US. It can be characterized by the overlap of the samples in the phase space, which can be quantitatively measured by, for instance, the overlap matrix proposed by Klimovich et al. [52]. As shown in Figure S1 in the Supplementary Materials, for both DA reactions, the overlap between neighboring windows are larger than 0.03, which is the lower-limit suggested by Klimovich et al. It indicates that the phase space overlap is sufficient for the subsequent MBAR analysis. Therefore, all the PM6/MM FE surfaces calculated by the MBAR method are statistically reliable. The other factor is the similarity between the PM6/MM Hamiltonian and the B3LYP/MM Hamiltonian, which determines the reliability of the weighted TP and can be measured by reweighting entropy [45]. As shown in Figure S2, overall reweighting entropy values are large enough for yielding reliable results in the weighted TP calculations. Because of the statistical noise in the wTP correction process, as shown in Figure S3, Gaussian processes regression (GPR) method [44] was used to smooth the FE surface after the wTP correction.

### 3.1. DA Reaction between CP and ACR

**Figure 3:**
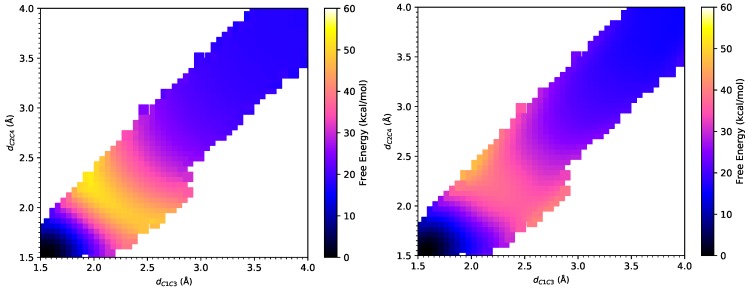
The free energy surfaces under the PM6/MM Hamiltonian (**left**) and under the B3LYP/MM Hamiltonian after Gaussian process regression (**right**) for Diels–Alder reaction between cyclopentadiene and acrylonitrile.

As shown in Figure 3, the product state defined by $d_{C1C3}=$ 1.58 Å and $d_{C2C4}=$ 1.58 Å is a reference state with zero free energy and the reactant state is defined with $d_{C1C3}=$ 4.00 Å and $d_{C2C4}=$ 4.00 Å. From the FE surfaces obtained by our method, the transition state can be located at $(\eta_{1},\eta_{2})=$ (2.41 Å, 1.92 Å) at the PM6/MM level, which is different from $(\eta_{1},\eta_{2})=$ (2.28 Å, 2.00 Å) at the PM3/MM level obtained by Jorgensen et al. and indicates stronger asynchronism of this reaction at the PM6/MM level than that at the PM3/MM level. Meanwhile, the transition state at the B3LYP/MM level is located at $(\eta_{1},\eta_{2})=$ (2.49 Å, 2.05 Å), which shows a small difference from that obtained at the PM6/MM level and also manifests the strong asynchronism of this reaction. As listed in Table 1, Jorgensen and coworkers showed that the activation free energy for this DA reaction is 34.0 ± 0.5 kcal/mol under PM3/MM Hamiltonian [7], and 24.7 kcal/mol under AM1/MM Hamiltonian [6]. In the current work, we found the activation free energy to be 30.9 ± 0.1 kcal/mol under PM6/MM Hamiltonian. These semi-empirical methods significantly overestimate the activation barrier for this DA reaction, which has an experimental value of 22.2 kcal/mol [3]. The large deviations in the activation barrier lie in the limited accuracy of these semi-empirical methods, as pointed out in Ref. [7]. An activation barrier of 20.5 ± 0.6 kcal/mol is obtained at the B3LYP/MM level via the weighted TP correction from the PM6/MM free energy landscape, which is much closer to the experimental value of 22.2 kcal/mol [3] than those semiempirical methods. It is interesting to note that B3LYP/IEF-PCM overestimates the activation free energy by 8.1 kcal/mol, whereas MP2/IEF-PCM underestimates the activation free energy by 4.6 kcal/mol. Both of them had worse performance than B3LYP/MM in the explicit solvent model. In terms of the reaction free energy, the B3LYP/MM method yielded a value of −15.7 ± 0.6 kcal/mol, which is much closer to the value of −15.8 kcal/mol at the MP2/IEF-PCM level, compared to −26.2 kcal/mol at the AM1/MM level, −16.7 ± 0.6 kcal/mol at the PM3/MM level and −17.5 ± 0.1 kcal/mol at the PM6/MM level. Surprisingly, B3LYP/IEF-PCM significantly underestimates the exothermicity of this reaction, which may come from an inadequate description of the solvent by the continuous solvent model.

As listed in Table 2, the projection of the solvent electric field on M1 (the midpoint of C1–C3 bond) has a magnitude of $-14.6\times 10^{-4}$ a.u. at the B3LYP/MM level. This electric field facilitates the charge transfer and stabilizes the transition state. As listed in Table 3, from the reactant to the transition state, the charge of C1 atom increases by 0.12*e*, and that of C3 atom decreases by 0.18*e*, which generates a large dipole moment change along this C1–C3 bond. The projection of the solvent electric field has a magnitude of $-19.7\times 10^{-4}$ a.u. on M2, which is even stronger than that along the C1–C3 bond. Although this electric field also facilitates the charge transfer from the diene to the dienophile, we observed an inverse flow of electron from C4 to C2. The charge of C2 atom decreases by 0.02*e*, and that of C4 increases by 0.09*e*, which also generates a dipole moment change along this C2–C4 bond. These two dipole–electric field interactions stabilize the transition state by about 0.4 kcal/mol in total.

### 3.2. DA Reaction between CP and NAP

**Figure 4:**
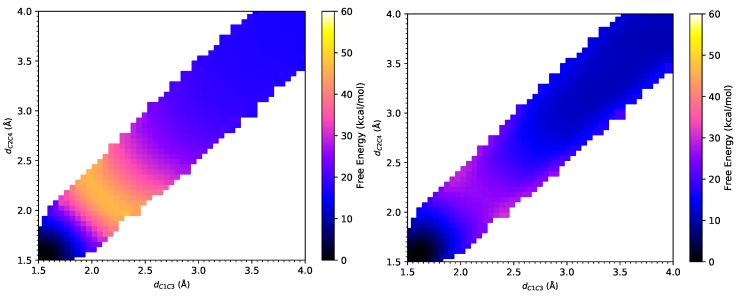
The free energy surfaces under the PM6/MM Hamiltonian (**left**) and under the B3LYP-D3/MM Hamiltonian after Gaussian process regression (**right**) for Diels–Alder reaction between cyclopentadiene and 1-4-naphthoquinone.

From the FE surfaces, as shown in Figure 4, the transition state can be located at $(\eta_{1},\eta_{2})=$ (2.14 Å, 2.18 Å) at the PM6/MM level, which has a slight deviation from $(\eta_{1},\eta_{2})=$ (2.25 Å, 2.22 Å) at the PM3/MM level obtained by Jorgensen et al. The transition state is located at $(\eta_{1},\eta_{2})=$ (2.23 Å, 2.19 Å) at the B3LYP-D3/MM level [53]. These small differences in these two RC come from statistical noise in the samples. PM3/MM agrees with B3LYP-D3/MM better than PM6/MM does. All the transition state positions obtained by different methods indicate a symmetrical and synchronous process of this reaction [7]. As listed in Table 1, the activation free energy for this DA reaction is 26.0 ± 0.5 kcal/mol under the PM3/MM Hamiltonian [7], and 27.6 kcal/mol under the AM1/MM Hamiltonian [6] according to Jorgensen et al. In contrast, our results was 29.6 ± 0.1 kcal/mol under the PM6/MM Hamiltonian. Thus, similar to the CP-ACR reaction, all semi-empirical methods again overestimate the activation barrier for this DA reaction, which has an experimental value of 16.6 kcal/mol [2]. Encouragingly, an activation barrier of 14.3 ± 0.7 kcal/mol at the B3LYP-D3/MM level was obtained via weighted TP from the PM6/MM free energy landscape, which is very close to the experimental value. With IEF-PCM, B3LYP-D3 overestimates the activation free energy by 4.1 kcal/mol, while MP2 significantly underestimates the activation free energy by 10.0 kcal/mol. In the meantime, B3LYP-D3/MM gives a reaction free energy of −11.5 ± 0.7 kcal/mol, which is also very close to the value of −13.9 kcal/mol calculated at the MP2/IEF-PCM level. Those from AM1/MM, PM3/MM and PM6/MM calculations are −4.4 kcal/mol, −20.1 ± 0.6 kcal/mol, and −16.7 ± 0.1 kcal/mol, respectively, showing larger deviations. Again, B3LYP-D3/IEF-PCM significantly underestimates the exothermicity of this reaction.

As listed in Table 2, the projection of the solvent electric field on M1 has a large magnitude of $-28\times 10^{-4}$ a.u. at the B3LYP-D3/MM level. This electric field facilitates the charge transfer and stabilizes the transition state. As listed in Table 3, from the reactant to the transition state, the charge of C1 atom increases by 0.09*e*, and that of C3 atom decreases by 0.05*e*. This local dipole moment stabilizes the transition state by interacting with the solvent-generated electric field. The projection of the solvent electric field on M2 has a magnitude of $-36.2\times 10^{-4}$ a.u., which is even stronger than that along the C1–C3 bond. The charge of C2 atom increases by 0.10*e*, and that of C4 decreases by 0.03*e*. This dipole moment also lowers the energy of the transition state in the electrostatic environment of the solvent molecules. These two dipole–electric field interactions stabilize the transition state by about 1.1 kcal/mol in total.

### 3.3. Computational Expense

The estimated wall-clock time for the computations of the QM/MM FE surfaces at the B3LYP/MM level are listed in Table 4. For the calculations of the QM/MM FE surfaces via weighted TP, the wall-clock time includes both the time for generating the SE QM/MM trajectories and the time for the single-point energy calculations at the B3LYP/MM level. The estimated wall-clock time for a direct free energy calculation at the B3LYP/MM level is also listed. It can be seen that the calculation efficiency is enhanced by about 139 and 376 times for CP/ACR and CP/NAP reactions, respectively, via this indirect free energy calculation utilizing a dual-Hamiltonian approach. Because one out every 1000 configurations are required for energy calculations at the ab initio level in this approach (1000 configurations for each 1-ns window in the indirect approach vs. 1,000,000 configurations in the direct approach), the efficiency enhancement can never exceed 1000. Besides, the computational cost at the PM6/MM level is non-negligible relative to the B3LYP/MM calculations for such small systems. It can also be seen that the efficiency enhancement is greater for the larger reaction system (CP/NAP), because the cost for the low-level sampling becomes less significant relative to that for the single-point energy evaluations at the *ai* level for larger molecules. Overall, an enhancement of two orders of magnitude in efficiency is quite satisfactory.

## 4. Conclusions

For the Diels–Alder reactions in solution, the computation of converged free energy (FE) surfaces at ab initio (*ai*) QM/MM level is still far from being affordable. In this work, we applied our recently proposed MBAR+wTP method to calculate the two-dimensional FE surfaces of two Diels–Alder reactions of cyclopentadiene with either acrylonitrile or 1-4-naphthoquinone at *ai* QM/MM level with much less computational expense. Due to some approximation lying in the semi-empirical (SE) method, the FE surfaces at SE QM/MM level deviate from experimental values by several kcal/mol in terms of the activation and the reaction free energies. However, our method can yield the FE profile at the *ai* QM/MM level without performing the expensive *ai* QM/MM MD simulations. Besides the FE surface, other ensemble-averaged properties such as the amount of charge transferred and the external electric potential/field are also readily available. The results agree much better with the experimental measurements than those obtained by other methods for these two Diels–Alder reactions in terms of the activation free energy. Care must be taken when using implicit solvent models, especially when calculating the properties of transition state. Further validation of this method to systems of much higher complexity such as enzymatic reactions will be carried out in future studies.

## Figures and Tables

**Table 1:** Activation free energy $\Delta G^{‡}$, reaction free energy ΔG (in kcal/mol) and the structure of the transition state at 298.15 K for the Diels–Alder reactions between cyclopentadiene and acrylonitrile (ACR), 1-4-naphthoquinone (NAP).

Dienophile	Method	$\Delta G^{‡}$	ΔG	$\left(\eta_{1},\eta_{2}\right){\;}^{a}$
ACR	PM3/MM ${}^{b}$	34.0 ± 0.5	−16.7 ± 0.6	(2.28, 2.00)
	AM1/MM ${}^{c}$	24.7	−26.2	
	PM6/MM	30.9 ± 0.1	−17.5 ± 0.1	(2.41, 1.92)
	B3LYP/MM	20.5 ± 0.6	−15.7 ± 0.6	(2.49, 2.05)
	B3LYP/IEFPCM	30.3	−2.69	(2.54, 2.04)
	MP2/IEFPCM	17.6	−15.8	(2.40, 2.18)
	Exp. ${}^{d}$	22.2		
NAP	PM3/MM ${}^{b}$	26.0 ± 0.5	−20.1 ± 0.6	(2.25, 2.22)
	AM1/MM ${}^{c}$	27.6	−4.4	
	PM6/MM	29.6 ± 0.1	−16.7 ± 0.1	(2.14, 2.18)
	B3LYP-D3/MM	14.3 ± 0.7	−11.5 ± 0.7	(2.23, 2.19)
	B3LYP-D3/IEFPCM	20.7	−2.62	(2.18, 2.18)
	MP2/IEFPCM	6.6	−13.9	(2.26, 2.26)
	Exp. *^e^*	16.6		

^*a*^ Transition state position in Å; ^*b*^ Ref. [7] by Jorgensen et al. using PDDG/PM3/MM/MC; ^*c*^ Ref. [6] with one-dimensional reaction coordinate; ^*d*^ Ref. [3] at 303.15 K; ^*e*^ Ref. [2].

**Table 2:** The projections of electric fields on points M1 and M2, respectively, along unit vectors ${\vec{e}}_{1}$ and ${\vec{e}}_{2}$ (in $10^{-4}$ a.u. where 1 a.u. = 51.42 V/Å) for the Diels–Alder reactions between cyclopentadiene and acrylonitrile (ACR), 1-4-naphthoquinone (NAP).

Dienophile	Method	Locations	Projection of E-Field
ACR	B3LYP/MM	M1	−14.6 ± 0.3
		M2	−19.7 ± 0.4
NAP	B3LYP−D3/MM	M1	−28.0 ± 0.5
		M2	−36.2 ± 0.7

**Table 3:** The Mulliken partial charges (in a.u.) of all atoms shown in Figure 2 for the Diels–Alder reactions between cyclopentadiene and acrylonitrile (ACR), 1-4-naphthoquinone (NAP).

Dienophile	Method	Atoms	Reactant State	Transition State	Product State
ACR	B3LYP/MM	C1	−0.13	−0.01	−0.05
		C2	−0.15	−0.17	−0.05
		C3	−0.18	−0.36	−0.13
		C4	−0.20	−0.11	−0.28
		R1	0.29	0.35	0.22
		R2	0.38	0.29	0.28
NAP	B3LYP−D3/MM	C1	−0.15	−0.06	−0.04
		C2	−0.14	−0.04	−0.03
		C3	−0.21	−0.26	−0.25
		C4	−0.23	−0.26	−0.26
		R1	0.30	0.34	0.26
		R2	0.44	0.29	0.32

**Table 4:** Estimated wall-clock time in a unit of hours for the computations of the QM/MM free energy surfaces at the B3LYP/6-31G(d) level. Assuming one node with 16 cores of Intel Xeon CPU E5-2660 2.20 GHz was used.

Dienophile	PM6/MM to B3LYP/MM Indirect			Direct B3LYP/MM ${}^{a}$
	Sampling	Energy Evaluation	Total	
ACR	5762	1459	7221	1,006,041
NAP	5945	3907	9852	3,704,062

^*a*^ Using the same number of windows as in the semi-empirical simulations, and one 1-ns simulation for each window.

## Supplementary Materials

The following are available online at http://www.mdpi.com/1420-3049/23/10/2487/s1. Figure S1: The overlap matrix elements at the PM6/MM level for the Diels–Alder reactions between: (a) cyclopentadiene and acrylonitrile; and (b) cyclopentadiene and 1-4-naphthoquinone.; Figure S2: The reweighting entropy values in reweighting from the PM6/MM Hamiltonian to the B3LYP(-D3)/MM Hamiltonian for the Diels–Alder reactions between: (a) cyclopentadiene and acrylonitrile; and (b) cyclopentadiene and 1-4-naphthoquinone.; Figure S3: The raw free energy surfaces under the B3LYP(-D3)/MM Hamiltonian for the Diels–Alder reactions between: (a) cyclopentadiene and acrylonitrile; and (b) cyclopentadiene and 1-4-naphthoquinone before Gaussian Process Regression.

## Abbreviations

The following abbreviations are used in this manuscript:

DA	Diels–Alder
FE	Free Energy
*ai*	ab initio
QM	Quantum Mechanical
MM	Molecular Mechanical
US	Umbrella Sampling
MD	Molecular Dynamics
SE	Semi-Empirical
MBAR	Multistate Bennett Acceptance Ratio
wTP	weighted Thermodynamic Perturbation
CP	Cyclopentadiene
ACR	Acrylonitrile
NAP	1-4-Naphthoquinone
FEP	Free Energy Perturbation
TS	Transition State
EVB	Empirical Valence Bond
SAMPL	Statistical Assessment of the Modeling of Proteins and Ligands
RC	Reaction Coordinate
GPR	Gaussian Processes Regression
RRHO	Rigid-Rotor/Harmonic-Oscillator
IRC	Intrinsic Reaction Coordinate
IEF-PCM	Integral Equation Formalism of the Polarizable Continuous Solvent Model
MP2	2nd-Order Møller–Plesset Perturbation Theory
AM1	Austin Model 1
PM3	Parameterization Method 3
PM6	Parameterization Method 6
