# Supplementary material for: Efficient Computation of Free Energy Surfaces of Diels–Alder Reactions in Explicit Solvent at Ab Initio QM/MM Level
Source: Molecules. 2018 Sep 28;23(10):2487. doi: 10.3390/molecules23102487 (PMC6222833; doi:10.3390/molecules23102487)
Supplement: Supplementary file 1 [file molecules-23-02487-s001.pdf]

Supporting Information
Efficient Computation of Free Energy Surfaces of Diels-Alder Reactions in Explicit Solvent at ab initio QM/MM Level

Pengfei Li,[†] Fengjiao Liu,[†] Xiangyu Jia,[†,‖] Yihan Shao,[‡] Wenxin Hu,[*,¶] Jun Zheng,[¶] and Ye Mei[*,†,§,‡]

†*State Key Laboratory of Precision Spectroscopy, School of Physics and Materials Science, East China Normal University, Shanghai 200062, China*

‡*Department of Chemistry and Biochemistry, University of Oklahoma, Norman OK 73019, United States of America*

¶*The Computer Center, School of Computer Science and Software Engineering, East China Normal University, Shanghai 200062, China*

§*NYU-ECNU Center for Computational Chemistry at NYU Shanghai, Shanghai 200062, China*

‖*Current address: NYU-ECNU Center for Computational Chemistry at NYU Shanghai, Shanghai 200062, China*

E-mail: wxhu@cc.ecnu.edu.cn; ymei@phy.ecnu.edu.cn

1 Reliability of the MBAR Analysis of the Trajectories from Two-Dimensional Umbrella Samplings

The reliabilities of the MBAR calculations are examined by calculating the overlap matrix proposed by Mobley et. al.,[1] which essentially measures the magnitude of the phase space overlap. The elements of the $S \times S$ overlap matrix are defined as[1,2]

$$\begin{aligned} O_{tt'} =& \sum_{i=1}^{S} \sum_{l=1}^{N_i} N_t w_t(\mathbf{x}_{i,l}) w_{t'}(\mathbf{x}_{i,l}) \\ =& \sum_{i=1}^{S} \sum_{l=1}^{N_i} \frac{N_t e^{-\beta\left[W_t(\mathbf{x}_{i,l})-f_t^{(b)}\right]} e^{-\beta\left[W_{t'}(\mathbf{x}_{i,l})-f_{t'}^{(b)}\right]}}{\left\{\sum\limits_{k=1}^{S} N_k e^{-\beta\left[W_k(\mathbf{x}_{i,l})-f_k^{(b)}\right]}\right\}^2}. \end{aligned} \tag{S1}$$

The overlap was computed for each pair of neighboring windows, which should be no smaller than 0.03 in order to ensure a reliable MBAR analysis as suggested by Klimovich et al.[1]

2 Reliability of wTP Calculations

It is well known that TP requires significant overlap in phase space between the sampled (PM6 in this work) Hamiltonian and the target (B3LYP in this work) Hamiltonian.[3] Because TP often suffers from numerical instability, the "reweighting entropy"[4] can be used to measure the reliability of wTP calculations.[2] It is defined as

$$\mathcal{S}(\boldsymbol{\eta}) = -\frac{1}{\ln\left(\sum\limits_{i=1}^{S}\sum\limits_{l=1}^{N_i}\delta(\boldsymbol{\eta}_{(i,l)}-\boldsymbol{\eta})\right)} \sum_{i=1}^{S}\sum_{l=1}^{N_i} \mathcal{P}(\boldsymbol{\eta}_{(i,l)}) \ln \mathcal{P}(\boldsymbol{\eta}_{(i,l)}), \tag{S2}$$

where

$$\mathcal{P}\left(\boldsymbol{\eta}_{(i,l)}\right) = \frac{w_L\left(\mathbf{x}_{\boldsymbol{\eta}_{(i,l)}}\right) e^{-\beta\left[U_H\left(\mathbf{x}_{\boldsymbol{\eta}_{(i,l)}}\right)-U_L\left(\mathbf{x}_{\boldsymbol{\eta}_{(i,l)}}\right)\right]}}{\sum\limits_{i=1}^{S}\sum\limits_{l=1}^{N_i} w_L\left(\mathbf{x}_{\boldsymbol{\eta}_{(i,l)}}\right) e^{-\beta\left[U_H\left(\mathbf{x}_{\boldsymbol{\eta}_{(i,l)}}\right)-U_L\left(\mathbf{x}_{\boldsymbol{\eta}_{(i,l)}}\right)\right]}}. \tag{S3}$$

Here, $\sum_{i=1}^{S}\sum_{l=1}^{N_i}\delta\left(\boldsymbol{\eta}_{(i,l)}-\boldsymbol{\eta}\right)$ represents the number of configurations falling into bin $\boldsymbol{\eta}$ among the samples from all the two-dimensional biased simulations. The larger the reweighting entropy is, the more reliable the TP calculation can be.

References

(1) Klimovich, P. V.; Shirts, M. R.; Mobley, D. L. Guidelines for the Analysis of Free Energy Calculations. *J. Comput. Aid. Mol. Des.* **2015**, *29*, 397–411.

(2) Li, P.; Jia, X.; Shao, Y.; Mei, Y. Computation of Free Energy Profile at *ab initio* QM/MM Level Made Orders of Magnitude Faster via the Reference-Potential Approach Using Weighted Thermodynamics Perturbation. arXiv.org, 2018; https://arxiv.org/abs/1801.03674.

(3) Lu, N.; Woolf, T. B. In *Free Energy Calculations*; Chipot, C., Pohorille, A., Eds.; Springer: Heidelberg, 2007; pp 199–247.

(4) Wang, M.; Li, P.; Jia, X.; Liu, W.; Shao, Y.; Hu, W.; Zheng, J.; Brooks, B. R.; Mei, Y. Efficient Strategy for the Calculation of Solvation Free Energies in Water and Chloroform at the Quantum Mechanical/Molecular Mechanical Level. *J. Chem. Inf. Model.* **2017**, *57*, 2476–2489.

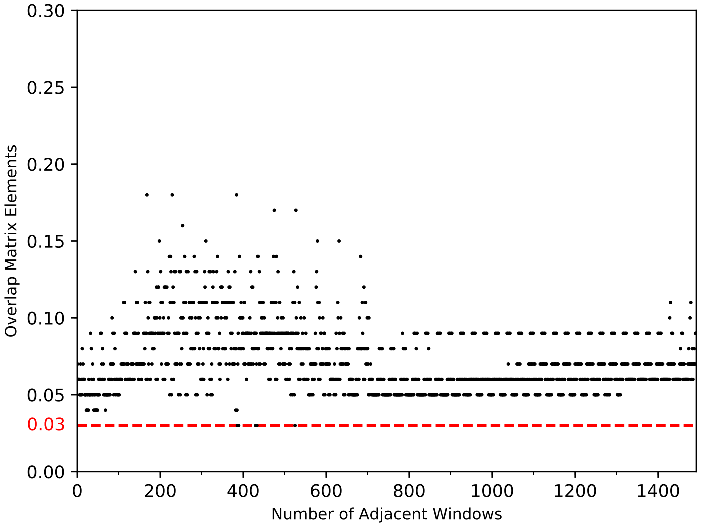

(a)

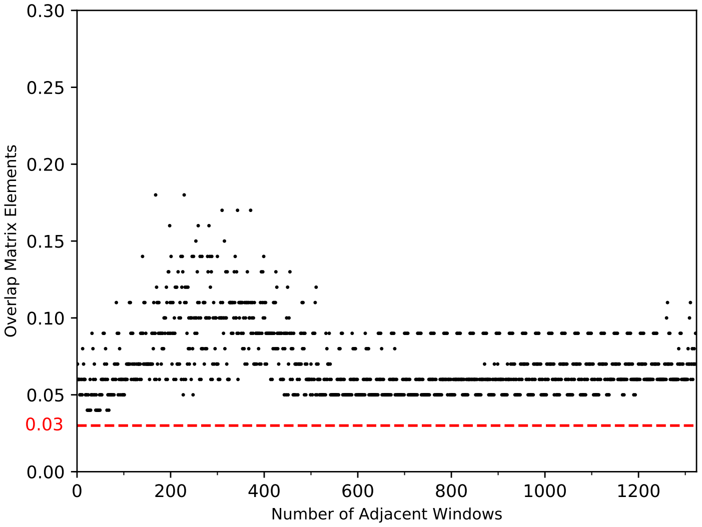

(b)

Figure S1: The overlap matrix elements at the PM6/MM level for the Diels-Alder reactions between cyclopentadiene and (a) acrylonitrile, (b) 1-4-naphthoquinone.

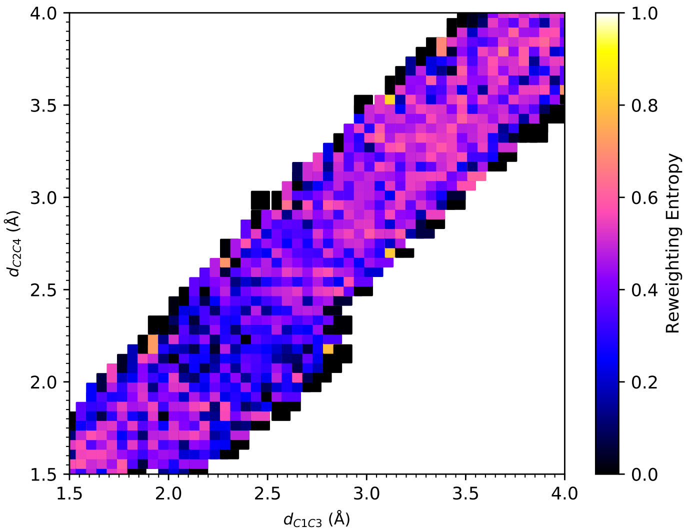

(a)

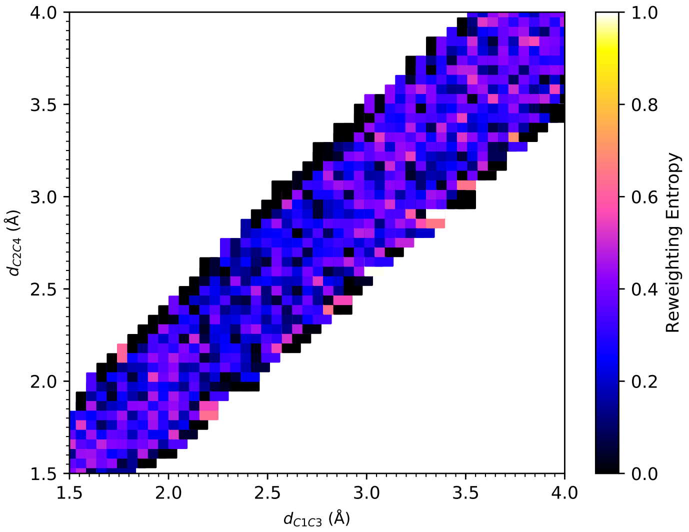

(b)

Figure S2: The reweighting entropy values in reweighting from the PM6/MM Hamiltonian to the B3LYP(-D3)/MM Hamiltonian for the Diels-Alder reactions between cyclopentadiene and (a) acrylonitrile, (b) 1-4-naphthoquinone.

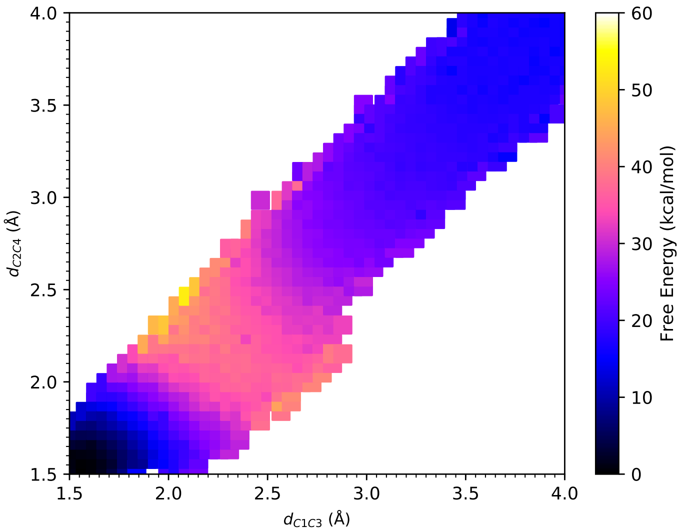

(a)

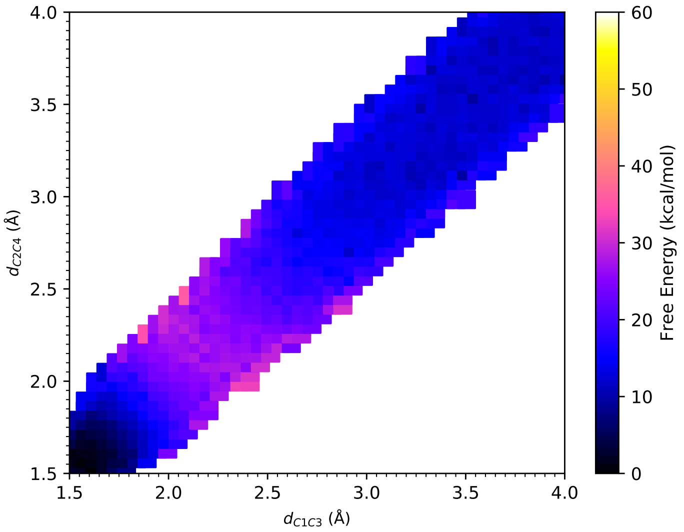

(b)

Figure S3: The raw free energy surfaces under the B3LYP(-D3)/MM Hamiltonian for the Diels-Alder reactions between cyclopentadiene and (a) acrylonitrile, (b) 1-4-naphthoquinone before Gaussian Process Regression.
